# Current Understanding of Pain Neurophysiology among Physiotherapists Practicing in Saudi Arabia

**DOI:** 10.3390/healthcare9091242

**Published:** 2021-09-21

**Authors:** Ahmed Alhowimel, Faris Alodiabi, Dalyah Alamam, Mazyad Alotaibi, Julie Fritz

**Affiliations:** 1 Department of Health and Rehabilitation Science, Sattam Bin Abdulaziz University, Al-Kharj 16278, Saudi Arabia; maz.alotaibi@psau.edu.sa; 2 Department of Health Rehabilitation Sciences, College of Applied Medical Sciences, King Saud University, Riyadh 11451, Saudi Arabia; falodaibi@ksu.edu.sa (F.A.); dalimam@ksu.edu.sa (D.A.); 3 Department of Physical Therapy and Athletic Training, University of Utah, Salt Lake City, UT 84112, USA; julie.fritz@utah.edu

**Keywords:** pain neuroscience, physiotherapy, knowledge

## Abstract

To ensure the effective management of patients’ pain, it is important that physiotherapists have a good understanding of the neuroscience behind pain. A major barrier to adequate pain management is that, for patients, there is limited access to clinicians who are knowledgeable about pain. This study examined the level of knowledge regarding pain neurophysiology among physiotherapists currently practicing in Saudi Arabia. Method: The study was a cross-sectional web-based survey that utilized the 12-item Revised Neurophysiology of Pain Questionnaire. Descriptive and inferential statistics were used to describe levels of knowledge regarding pain neurophysiology and to examine differences in knowledge based on the characteristics of the participating physiotherapists (gender, educational level, experience, practice region, and country where their highest educational level was attained). Results: One hundred and eleven physiotherapists (58.6% male) from various regions and educational backgrounds participated in the study. Out of a maximum Revised Neurophysiology of Pain Questionnaire score of 12, the mean ± standard deviation (SD) was 6.7 ± 2.2; 90% of physiotherapists scored 9 (75%) or less. None of the examined characteristics of the participants were associated with knowledge. Conclusion: Physiotherapists in Saudi Arabia showed limited knowledge of the neurophysiology of pain; however, this was not related to the personal characteristics that were examined. The continuation of education in modern pain science is recommended for physiotherapists, especially those dealing with patients suffering from chronic pain. Clinical Relevance: The physiotherapists who took part in this study displayed limited knowledge of pain neuroscience; this limited knowledge might suggest the need for a more bio-anatomical approach to pain management. There is a need for tailored medical education to address pain neuroscience knowledge in current physiotherapist practitioners.

## 1. Introduction

Pain is a common feeling that can accompany different illnesses. Pain does not necessarily indicate tissue damage, and much can be learned about it from recent literature [1,2,3]. Regrettably, information on recent advances in pain neurophysiology and modern pain science may not be delivered well to healthcare workers. Improper management of and education about chronic pain may lead to persistent pain and disability in specific individuals [4]. Therefore, recognition of the importance of ensuring a sound understanding of pain neurophysiology among healthcare workers dealing with pain is highly relevant.

One approach to managing chronic pain is pain neurophysiology education, i.e., clarifying the patient’s understanding of neurophysiological concepts of pain to lessen the degree of threat (for example, explaining that pain and tissue damage are not equivalent) [5,6,7,8]. Pain neurophysiology education has some similarities with cognitive behavioral therapy. The technique aims to challenge and restructure patients’ inappropriate beliefs to change consequent behavior and promote positive coping strategies that enhance recovery [9]. However, to properly guide patients in pain management, health professionals themselves must have a basic knowledge of modern pain neuroscience [6,10,11,12].

Numerous publications have suggested that pain science education has not been updated in some countries [13,14,15,16,17]. For instance, Alodaibi et al. [12] reported that although final-year physical therapy students in Saudi Arabia demonstrated higher levels of knowledge in pain science than those at the beginning of their undergraduate degree, the effect size was small and probably not clinically meaningful. This finding suggests that physiotherapy (PT) education in Saudi Arabia has not adequately incorporated recent discoveries reported in the pain literature (i.e., modern pain science). This has created an opportunity to investigate whether pain neurophysiology understanding evolves with self-learning, continuous education, and clinical practice.

Previous studies showed that the prevalence of chronic pain ranged from 19.0% to 46.4% in a sample of Saudi participants residing in various regions of Saudi Arabia [18,19]. In two studies, back pain was the most prevalent form of pain reported. Chronic pain disorders are highly prevalent in Saudi Arabia, placing a burden on the healthcare system, and it is worth investigating physical therapists’ current knowledge of pain neurophysiology in order to improve our understanding of the management (including the educational aspects) of cases of pain. Therefore, our aim in this study was to explore the knowledge level of pain science among PTs practicing in Saudi Arabia and to identify any factors that may affect therapists’ levels of knowledge.

## 2. Methods and Materials

A cross-sectional survey design was used to examine pain neuroscience knowledge among PTs practicing in Saudi Arabia. The Institutional Review Board of the Ministry of Health approved the study (IRB: 2019-0060E).

### 2.1. Data Collection

#### 2.1.1. Participants

Eligible participants in this study were Saudi physiotherapists working in Saudi Arabia (*N* = 1618).

#### 2.1.2. Procedure

Survey data were collected using an online survey platform, SurveyMonkey (www.SurveyMonkey.com) (accessed on 20 August 2019). A letter of invitation that included the aim of this study and the survey link was distributed through social media applications, including Twitter and WhatsApp, directly to PTs in Saudi Arabia. Participants responded online, and the survey platform logged the responses and added them to a results database. Four reminder notices were sent to redistribute the invitation letter at 1-month intervals. The questionnaire was open to responses from 8 May 2019 to 20 August 2019.

Consenting PTs completed a demographic section, which included their age, gender, years of professional experience, and educational qualifications. The participants also completed a section that included the pain neuroscience questionnaire.

The Revised Neurophysiology of Pain Questionnaire (RNPQ).

The questionnaire consists of 12 statements about the neurophysiology of pain. These statements assess the understanding of pain biology and physiology based on current pain science [5,20]. Each statement can be answered with ‘true’, ‘false’, or ‘undecided’. For each correctly answered item, 1 point is given; therefore, the total score ranges from 0 (worst knowledge) to 12 (best knowledge). A Rasch analysis of psychometric properties found the questionnaire to be acceptable for use as an assessment of an individual’s understanding of pain mechanisms [20]. We used the English version of the questionnaire, as English is the primary language used by PTs clinically and academically.

#### 2.1.3. Sample Size Calculation

In 2018, the Saudi Commission for Health Specialties (SCFHS) stated that the total number of registered/licensed Saudi PTs was 1618 [21].

The required sample size was calculated by setting the statistical power at an 80% CI, with a population size of 1618 and a margin of error of 5%. Thus, the required sample size for this study was 149 participants.

#### 2.1.4. Analysis

Statistical Package for the Social Sciences (SPSS; version 24) (IBM, Armonk, NY, USA) software was used to analyze the data. The means with standard deviation (SD), frequencies, and percentages were calculated in the descriptive analyses. One-way analysis of variance (ANOVA) tests and *t*-tests (independent samples) were used to analyze significant differences in scores based on different characteristics of the PTs (i.e., professional experience, gender, education level, the country where their highest educational level was attained, and region of practice). The scores for those who obtained their highest-level degree in Saudi Arabia versus other countries were also compared. The significance level was set at *p* ≤ 0.05.

## 3. Results

### 3.1. Demographics

A total of 111 PTs practicing in Saudi Arabia participated in the study, of whom 58.6% were male. Just over half (58.6%) were employed as a PT 1 (a PT with a bachelor’s degree in the current Saudi healthcare system grading), while the remainder were PT 2 (practicing PTs with a master’s degree, 29.7%) and consultants (practicing PTs with a doctoral degree, 11.7%). Of those questioned, 82.8% were working in government-run or private hospitals and clinics, and 17.8% were working in an academic/university environment. Most participants were aged between 26 and 40 years (74.8%) and had between 0 and 15 years of experience (89.2%) in this area of practice. Just over half (51.4%) of the participants were practicing in the Riyadh area (Table 1).

### 3.2. RNPQ Scores

**Figure 1:**
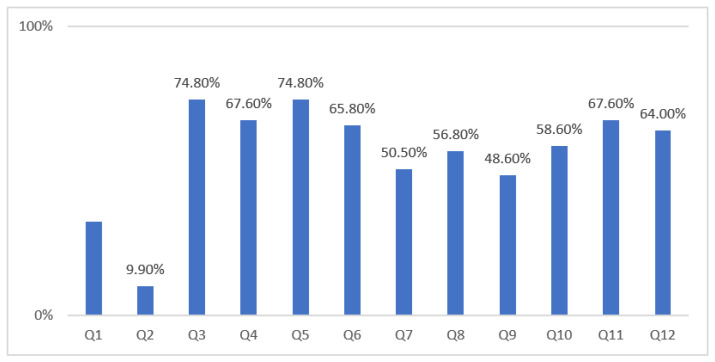
Percentage of correct answers for each of the 12 items on the Revised Neurophysiology of Pain Questionnaire.

The mean ± SD score on the RNPQ based on the entire sample was 6.7 ± 2.2. Scores ranged from 2 to 12; 10% of participants scored 10 or more. The items least often answered correctly (<50% correct answers) were item 1, ‘It is possible to have pain and not know about it’; item 2, ‘When part of your body is injured, special pain receptors convey the pain message to your brain’; and item 9, ‘Descending neurons are always inhibitory’. By contrast, item 3, ‘Pain only occurs when you are injured or at risk of being injured’, and item 5, ‘Special nerves in your spinal cord convey “danger” messages to your brain’, attained the highest correct scores, both with 74.8% correct answers (Figure 1).

### 3.3. RNPQ Scores Based on PTs’ Personal and Professional Characteristics

No significant difference in RNPQ score was found between the two genders (*p* = 0.61; 6.8 ± 2.3 for men and 6.6 ± 2.1 for women; Table 2). Likewise, no significant differences were found between groups based on the other characteristics examined, including the level of education (*p* = 0.43), the country where participants had acquired their highest degree (*p* = 0.16), and level of experience (*p* = 0.93) (Table 2). The scores of the PTs who obtained their highest educational degree in Saudi Arabia (6.4 ± 2.1) were not significantly different from the scores of those who obtained their highest qualification in other countries (7.2 ± 2.3; *p* = 0.09).

## 4. Discussion

This study aimed to assess knowledge of pain neurophysiology among PTs in Saudi Arabia and to examine factors that might explain differences. On average, PTs practicing in Saudi Arabia exhibited limited knowledge of the neurophysiology of pain. The understanding of the neurophysiology of pain for the PTs practicing in Saudi Arabia was similar across different settings and various professional and demographic characteristics, including gender.

A limited number of studies have been conducted to examine pain neurophysiology knowledge among PTs practicing worldwide. Generally, our results showed a reasonable level of knowledge among PTs in Saudi Arabia, as shown by an average score of 6.7 (55.8% correct answers) on the RNPQ. This level of knowledge was comparable to that in other studies using the RNPQ. For example, our score was similar to a previous study among Saudi PT students, who had a mean score of 6.2 (51.7%) using the same questionnaire [12] and similar to the score of South African PT students in their final year (58%) [22]. Furthermore, the score in the present study was comparable to the 55% score reported for untrained healthcare professionals (PTs, occupational therapists, psychologists, and rehabilitation counselors) in Australia [5]. However, the score was lower than that of Portuguese and Spanish PT students in their final year who attended formative active teaching sessions to improve their knowledge of pain neurophysiology. Those students scored 62.5% [23] and 68.92% [11], respectively, on the RNPQ.

The sample of PTs in the present study did not perform as well as PTs trained in the neurophysiology of pain, who scored 78% in one study [24]. This finding highlights the importance of educational approaches that aim to educate healthcare providers about pain neuroscience and have been shown to be helpful in managing chronic pain conditions [25,26]. Reportedly, PTs’ RNPQ score can improve significantly after two days of training [27,28,29,30,31].

The current study results also revealed that a high percentage of PTs gave correct responses to most of the RNPQ questions, except for three questions. Items 1, 2, and 9 only had a small percentage of correct answers (32.4%, 9.9%, and 48.6%, respectively). These questions involve the mechanism of nociception and pain modulation [32]. These results highlight the need to integrate pain neuroscience as a part of PTs’ educational and training programs and could be beneficial in practice [27,28,29,30]. Accurate knowledge of pain neurophysiology among healthcare professionals has been reported as essential and can reduce unhelpful pain-related beliefs and attitudes [33]. For example, an improvement in health professionals’ knowledge of pain biology was associated with the following impacts on patients with chronic pain: reductions in pain, pain-catastrophizing, and fear-avoidance behaviors, and an improvement in function [33,34]. Appropriate pain neurophysiology knowledge is essential; it may positively influence patients’ beliefs about the causes and consequences of chronic pain and help in the management of chronic pain conditions [6,33,35]. Characteristics such as gender, professional experience, and educational level did not seem to have an impact on the pain neurophysiology knowledge of PTs in our study. The region of practice in Saudi Arabia was the only significant factor, with a mean RNPQ score of more than seven for PTs in eastern, northern, and central regions. The number of participants from eastern and northern regions was low and selection bias may have inflated the scores. The central region is closer to the major universities, and thus PTs in this region may have relatively higher knowledge. Longitudinal analyses to examine the influence of different educational levels and other demographic and clinical characteristics, and their impact on pain knowledge, is worthy of further investigation among healthcare providers. Furthermore, the influence of training and educational programs about pain neurophysiology among PTs and how these might influence patients’ beliefs in, for example, movement-related fear and pain catastrophizing [34], has not yet been explored in the Saudi Arabian context. For this reason, it would be sensible to explore whether training or educational classes could improve PTs’ knowledge and influence the beliefs and attitudes of their patients with chronic pain.

The results of this study should be acknowledged with the consideration of some limitations. There is no determined cut-off score that represents sufficient knowledge; therefore, the scores and percentages in this study were compared with previous data. However, the RNPQ is still a valuable tool for assessing the conceptualization of the biological processes behind pain, as well as for evaluating the impact of cognitive treatments in clinical practice and research with acceptable psychometric characteristics [20]. Furthermore, the low number of participants and selection bias limit the generalizability of the results. The study only recruited 75% of the number of participants needed, despite efforts to recruit the targeted number. Participants in this research, on the other hand, worked in various healthcare sectors and regions across Saudi Arabia. Furthermore, more than 37% of the sample obtained their undergraduate education outside of Saudi Arabia. Finally, one of the main goals of the education of healthcare practitioners in pain science is to change patient behaviors by lessening the fear and avoidance that arise through patients’ association of pain with movement. However, this study did not measure fear or other related factors, so it is recommended that future studies examine the association between pain neurophysiology knowledge and other behavioral and practice aspects after pain education sessions with a sample size that is large enough.

### Clinical Implications

The findings of this study support the need to implement undergraduate curricula and/or postgraduate continuous learning courses on pain neurophysiology. This strategy could have extended implications for patients with chronic pain, potentially improving evidence-based pain management.

## 5. Conclusions

This study provides insight into the knowledge of pain neurophysiology among physical therapists practicing in Saudi Arabia. In general, PTs showed limited knowledge of the neurophysiology of pain. Additionally, no significant difference in the knowledge of pain neurophysiology was found across various characteristics.

## Figures and Tables

**Table 1:** Personal and professional characteristics of physical therapists.

Variabe	Category	*n*	%
Gender	Male	65	58.6
	Female	46	41.4
Age	20–25	16	14.4
	26–30	35	31.5
	31–35	25	22.5
	36–40	23	20.7
	41–45	4	3.6
	46–50	7	6.3
	Above 50	1	0.9
Job title	Physical therapist I	65	58.6
	Physical therapist II	33	29.7
	Consultant	13	11.7
Level of education	BSc	57	52.3
	MSc	34	29.7
	PhD	16	14.4
	DPT	4	3.6
Country from which the highest degree qualification was acquired	Saudi Arabia	68	62.2
	US	14	12.6
	UK	19	16.2
	Australia	2	1.8
	Other	8	7.2
Workplace	Governmental hospital/clinic	65	55.1
	Academia/university	21	17.8
	Private hospital/clinic	32	27.1
Years of experience	Less than 2 years	27	24.3
	2–5	28	25.2
	6–10	18	16.2
	11–15	26	23.4
	16–20	4	3.6
	More than 20 years	8	7.2
Province/region of practice	Central region (Riyadh)	57	51.4
	Central region (out of Riyadh)	11	9.9
	Northern region	3	2.7
	Western region	19	17.1
	Eastern region	10	9.0
	Southern region	11	9.9

**Table 2:** Scores on the Revised Neurophysiology of Pain Questionnaire, by participants’ personal and professional characteristics.

Variabe	Category	*n*	Mean ± SD	*p* *
Sex	Male	65	6.8 ± 2.3	0.61
	Female	46	6.6 ± 2.1	
Level of education	BSc	58	6.4 ± 2.1	0.43
	MSc	33	7.1 ± 2.0	
	PhD	16	7.1 ± 2.8	
	DPT	4	6.3 ± 2.2	
Country from which the highest degree qualification was acquired	Saudi Arabia	69	6.5 ± 2.1	0.16
	US	14	7.6 ± 2.0	
	UK	18	6.6 ± 2.6	
	Australia	2	9.5 ± 0.7	
	Other	8	7.0 ± 2.0	
Province/region of practice	Central region (Riyadh)	57	7.2 ± 2.4	0.05
	Central region (out of Riyadh)	11	6.0 ± 1.8	
	Northern region	3	7.3 ± 1.2	
	Western region	19	5.8 ± 1.9	
	Eastern region	10	7.5 ± 1.8	
	Southern region	11	5.6 ± 1.4	
Years of experience	Less than 2 years	27	6.5± 1.5	0.93
	2–5	28	6.9± 2.5	
	6–10	18	6.7± 2.3	
	11–15	26	6.8± 2.1	
	16–20	4	5.8± 2.2	
	More than 20 years	8	6.7± 3.5	

* Statistically significant using one-way analysis of variance (ANOVA) tests and *t*-tests.

## Data Availability

The datasets used and/or analyzed during the current study are available from the corresponding author upon reasonable request.
